# The influence of negative events on adolescents’ mobile phone addiction: the chain mediating role of personality traits and emotional regulation style

**DOI:** 10.3389/fpsyt.2025.1530212

**Published:** 2025-03-07

**Authors:** Mingxin Ji, Yi Qi, Huixin Tu, Siyi Wu, Xingrui Wang

**Affiliations:** School of Economics and Management (School of Accounting), Yunnan Minzu University, Kunming, China

**Keywords:** negative events, mobile phone addiction, personality traits, emotional regulation style, chain mediation

## Abstract

**Introduction:**

Teenagers’ excessive dependence on mobile phones has aroused widespread concern in society. However, the complex mechanisms underlying the relationship between negative events and adolescent mobile phone addiction have not been thoroughly studied. The study aims to delve into the specific relationship between negative events and adolescent mobile phone addiction, and further explore the mediating role of personality traits (neuroticism) and emotional regulation (expression inhibition) in this relationship.

**Methods:**

This article is based on an empirical study involving 1399 randomly selected survey questionnaires from ordinary higher education institutions in Jiangsu and Zhejiang regions of China. The Adolescent Life Events Scale is employed to assess the impact of negative events on adolescents. The College Student Mobile Phone Addiction Scale is utilized to explore adolescent mobile phone usage behavior and addiction tendencies. The Big Five Personality Questionnaire is used to analyze personality tendencies, while the Emotion Regulation Scale is used to evaluate the frequency of using emotion regulation strategies.

**Results:**

(1) A notable positive correlation exists between negative events and mobile phone addiction. (2) Neurotic personality and expression inhibition play a mediating role in the chain effect between negative events and mobile phone addiction.

**Discussion:**

This study enriches relevant research on adolescent mobile phone addiction and offers a guide for educators and parents to solve the issue of adolescent mobile phone addiction. Negative events are positively correlated with mobile phone addiction, and neurotic personality and expressive inhibition play a chain mediating role between them.

## 1 Introduction

By 2024, Internet citizen has an increase of 7.42 million compared to December 2023 in China. Teenagers are an important group of Internet users (1). Mobile phones are portable electronic devices that integrate communication, entertainment, office, and other functions, giving them a vital role in modern society. However, the accompanying negative effects of mobile phones, specifically, numerous scholars have been drawn to the matter of excessive reliance on mobile phones. Mobile phone addiction refers to mental and behavioral abnormalities caused by the repeated use of a single or multiple Internet related mobile phone functions. Mobile phone addiction is characterized by withdrawal symptoms when the use of mobile phones is reduced or completely stopped, and it manifests as a strong desire for reuse, accompanied by mental and physical discomfort (2). Mobile phone addiction among adolescents has escalated into a significant public health concern, with high school and college students are the groups most likely to experience mobile phone addiction (3, 4). In high school, the prefrontal lobe (rational center) is not mature, whereas the nucleus accumbens (pleasure center) is relatively developed. This physiological characteristic makes high school students more likely than other age groups to experience pleasure from using mobile phones, leading to mobile phone addiction (5). At university, college students’ learning tasks change, and they begin to construct their individual, collective, and rational selves. A lack of self-construction may lead to mental health problems and addictive behaviors (6–8).

Within the domain of addiction behavior research, negative life events constitute a crucial factor contributing to individual mobile phone dependence (9). Individuals experiencing such events frequently feel lonely, suffer from a deficiency in the sense of achievement and a lack of self confidence, with more severe cases potentially suffering from depression (8, 10). Consequently, they resort to the functions of mobile phones to fulfill their unmet psychological needs, thereby getting trapped in mobile phone addiction (4). Scholars have discovered that psychological factors such as “mindfulness”, “perception of social support”, can effectively alleviate the negative influence of adverse events on mobile phone addiction (9, 11). Mindfulness, as a technique for regulating attention and managing emotions, enables individuals to maintain awareness and acceptance of their current experiences, thereby reducing their dependence on mobile phones (11). The perception of social support refers to the extent to which an individual perceives social support. Such perception can offer emotional solace to individuals and alleviate the psychological stress caused by negative events (9). However, concurrently, psychological and emotional disorders such as “social anxiety”, “stress”, “relative deprivation”, “depression” and “alexithymia” may intensify an individual’s propensity for mobile phone addiction (9, 12–14). Alexithymia, on the other hand, refers to the difficulty individuals encounter in recognizing, understanding, and expressing emotions. This disorder may lead individuals to be more inclined to use external stimuli such as mobile phones to evade or relieve emotional distress when confronted with negative events (12). Social anxiety causes individuals to feel uneasy in interpersonal interactions and may thereby seek refuge in mobile phones (13). Stress and relative deprivation may result in individuals feeling dissatisfied and unfair, thereby augmenting their reliance on mobile phones (9, 14).Depressive moods may cause individuals to lose interest in daily activities and become more engrossed in the mobile phone world (10). Although existing studies have explored the relationship between negative events and mobile phone addiction, the majority have concentrated on surface level emotional responses, while the exploration of the underlying mechanisms is still inadequate. For example, how do negative events exert differential impacts on individuals? What are the underlying influential factors? Why do some individuals sink into depression after encountering negative events, using mobile phones as emotional comfort and becoming addicted, while others maintain an optimistic and positive attitude? Thus, this article will focus on personality traits and emotion regulation strategies as mediating variables to deeply probe into the more fundamental causes behind mobile phone addiction behavior. By exploring the mediating role of personality traits and emotion regulation strategies between negative events and mobile phone addiction, it aims to reveal the sources of individual differences and provide novel perspectives and strategies for the prevention and intervention of mobile phone addiction. This study examined mobile phone addiction with high school and college students as participants, explored the factors and corresponding internal mechanisms of negative events affecting mobile phone addiction, provided theoretical support for the formulation of scientific and reasonable intervention measures for mobile phone addiction, and offered suggestion for timely interventions by educators and parents. The marginal contribution of this article maybe two folds: (1) This article introduces the chain mediation effect model as an empirical research method, which not only demonstrates the superposition effect of different variables on the impact path, but also highlights the mediation effect characteristics between variables, breaking the single mediation model in traditional research and providing effective model support for the mediation mechanism of the complex correlation between the influence of negative events on mobile phone addiction in this article. (2) Through a systematic review of relevant literature at home and abroad, conducts a comprehensive analysis of two core dimensions underlying emotional responses personality traits and emotion regulation strategies, and endeavors to explore how these two factors affect and function in the complex relationship between negative events and mobile phone addiction. The study aims to offer more incisive insights and more effective strategic frameworks for the efficient prevention and intervention of mobile phone addiction issues. Simultaneously, it also injects new theoretical perspectives and practical wisdom into academic research in the field of mental health.

## 2 Research hypothesis

### 2.1 Negative events and mobile phone addiction

Negative events refer to events that can trigger negative emotional experiences, which promote the negative development of individual emotions. Such events have an important impact on the healthy development of the body and mind of individuals, especially those with weak psychological endurance, these negative events may have disastrous consequences (15). In psychology and sociology, negative events and negative life events are often used synonymously, as both refer to life experiences that adversely affect individuals. Given these research needs, this study selected a series of representative negative life events for in depth analysis. Existing empirical research results have revealed the mechanism of negative events’ influence on addictive behaviors (16), such as mobile phone, Internet, and short video use (9, 13, 17). In the study of addiction phenomena, negative events are regarded as a crucial risk factor (9). When individuals are exposed to persistent stressful stimuli, they might seek means to evade reality and alleviate anxiety (4). Smartphones, as omnipresent communication and entertainment tools in modern society, have become the first choice for many as an escape method due to their convenience, immediacy, and multifunctionality. Through indulging in mobile games, social media, et al. (9), individuals can temporarily forget the troubles in real life and gain transient pleasure and a sense of satisfaction (16). The General Stress Model suggests that individuals who have experienced more negative life events tend to relieve their stress through deviant or addictive behaviors (18, 19). The Internet Compensation Theory indicates that when individuals encounter set backs or difficulties in real life, they may seek compensation in the online world. Smartphones, as an important entrance to the online world, provide individuals with a platform to escape reality, seek comfort, and gain a sense of satisfaction (20). Nevertheless, such escape behavior is often accompanied by the risk of addiction, and once dependence is formed, it becomes extremely difficult to extricate oneself (16). This article draws on the classification of the Adolescent Life Events Scale developed by Liu et al. and later verified by Xin et al. (21). The scale divides life events into six categories: five typical negative events and other events. Since other events have no practical significance for subsequent research in this article, other events are excluded to further divide the negative events studied in this article into five categories: punishment, loss, interpersonal pressure, learning stress, and adaptation problems (Hereinafter referred to as the five categories) (21). The objective is to investigate the differential influence of various negative events on student group mobile phone dependency in depth, in order to propose specific intervention strategies.

Accordingly, this article presents the following hypotheses:

H1: A notable positive correlation exists between negative events and mobile phone addiction.

### 2.2 Mediating role of personality traits

Negative events, such as interpersonal conflicts and economic losses, often create an overflowing negative emotional environment. In this environment, individuals must mobilize their psychological resources to cope with and regulate their emotions. At this time, how to perceive and make decisions and choices accordingly is crucial to a people’s physical and mental well being (21). It is worth noting that individuals with neurotic personality traits tend to show more sensitive and fragile emotional responses when faced with negative events. They pay too much attention to and immerse themselves in negative emotions, making it difficult to effectively regulate and recover from emotions (22). Moreover, there is an additive effect between negative events and negative emotional experiences (23). When individuals with a high neuroticism score also experience more negative events, their susceptibility to negative emotions makes them more likely to experience emotional distress when faced with negative events.

Individuals with neurotic personality traits have a high propensity for mobile phone addiction (24).Specifically, people with neurotic personality traits may be more inclined to seek comfort, escape from reality, or meet certain psychological needs through mobile phone networks because of their internal emotional instability and overreaction to external stimuli, thus, they are prone to over dependence on mobile social networks. In conclusion, this article holds that individuals with high neuroticism tend to have difficulty controlling their emotions, possess stronger dependency and a tendency to escape from reality. When confronted with negative events, they may be more prone to getting trapped in negative emotions and find it hard to extricate themselves, thus seeking out media such as mobile phones as a means to escape reality.

Accordingly, this article presents the following hypotheses:

H2: Neuroticism mediates the relationship between negative events and mobile phone addiction.

### 2.3 Mediating role of emotional regulation

Emotional regulation involves the process by which individuals consciously or unconsciously change their emotional state. There are two main emotional regulation strategies based on different neural bases: cognitive reappraisal and expression inhibition (25, 26). The former as a prefocus strategy, is characterized by the reinterpretation of emotional events in a more positive or rational direction before an individual produces an emotional response. By adjusting the understanding and cognitive framework of emotional events, this strategy can reduce subjective unhealthy emotions and effectively boost the experience of positive feelings (26). Expression inhibition is a reaction oriented strategy aimed at the post emotional arousal stage. After an individual’s emotions are activated, they attempt to reduce the intensity of subjective emotional feelings by curbing the emotional expression behavior that is about to be displayed. However, the practical effect of expression inhibition is limited, it is difficult to effectively reduce the depth of emotional experience and the degree of physiological arousal (27). When individuals experience negative events, their adaptive and non adaptive cognitive emotion regulation strategies show a significant change trend (28). when adolescents experience more serious negative events, they are often more inclined to adopt negative expression inhibition strategies, which both aggravate their psychological burden and induce addictive behaviors (29). Individuals who exhibit expressive suppression tend to have difficulty in effectively dealing with their emotions when facing negative events (30), resulting in emotional repression and accumulation and potentially generating a sense of isolation from others emotionally, reducing emotional communication and real life interaction with others (31). Owing to the immediacy of modern mobile phones, individuals can promptly obtain satisfaction in aspects such as information, entertainment, and socialization. This immediate gratification effect may incline individuals to utilize mobile phones to evade negative events and emotional disturbances in reality. In the long run, this immediate gratification effect might gradually intensify an individual’s mobile phone usage behavior, ultimately leading to mobile phone addiction (13). Simultaneously, some research results show that emotional self regulation ability has a direct impact on mobile phone addiction, and a lack of emotional regulation ability may cause individuals to tend to seek solace through mobile phones when facing pressure, thus aggravating the potential danger of mobile phone addiction (18).

Accordingly, this article presents the following hypotheses:

H3: Emotional regulation mode (expression inhibition) mediates the relationship between negative events and mobile phone addiction.

### 2.4 Chain mediating effect of personality trait and emotion regulation style

Emotional regulation strategies are closely related to individual personality characteristics, emotional response tendencies, cognitive development, and social growth (12, 32). Individual personality traits can profoundly shape people’s emotional regulation mode, thus creating a notable influence on individual subjective happiness and emotional experiences (33). Specifically, different personality traits tend to predispose individuals to adopt specific emotion regulation strategies. For example, after encountering adverse life events in real life situations, individuals with neurotic personality traits more frequently employ expression inhibition as a means of emotion management (34). However, individuals who habitually adopt expression inhibition strategies often indicate that they will experience and display more negative emotional experiences that are difficult to convey through their bodies or language (35). In this negative feedback loop, the emotional accumulation and suppression caused by the high neurotic individual’s tendency to inhibit emotional expression may eventually erupt in a more intense form, exacerbating their negative emotional experience (12, 28). The ease and comfort provided by mobile communication have made it a haven for many people facing discomfort and difficulties in actual interpersonal communication, so they are tending to favor using mobile phones as a means of seeking social support and emotional solace (12).

Accordingly, this article presents the following hypotheses:

H4: Neurotic personality and expression inhibition mediate the relationship between negative events and mobile phone addiction.

**Figure 1:**
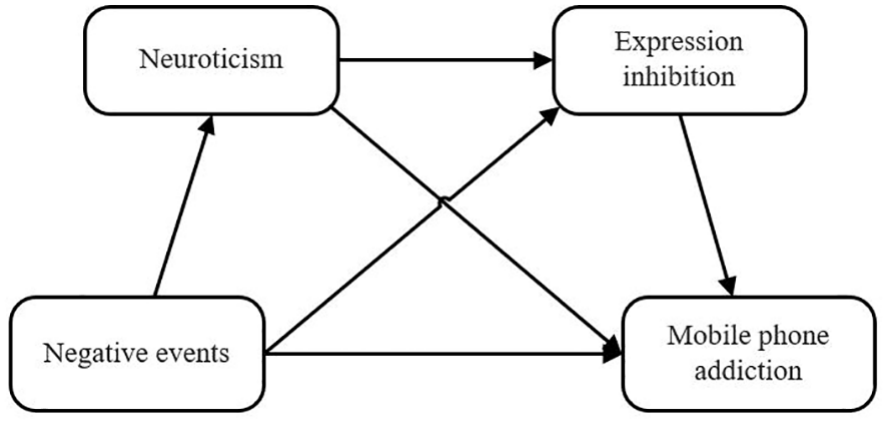
Negative events and mobile phone addiction chain mediation model.

Negative events and mobile phone addiction chain mediation model as show in **Figure 1**.

## 3 Methods

### 3.1 Participants and procedure

This research adopted a random cluster sampling method, and all participants voluntarily participated in the survey without any compensation provided to them. The study’s sample consists of teenagers in the Jiangsu and Zhejiang regions, which are areas with relatively high levels of economic development in China. The living standards of residents are relatively good, and the popularity of mobile phones among adolescents is high. This provides a rich sample for the study. Secondly, the two provinces have abundant educational resources and numerous high quality schools, which provides a good educational background for the study and helps to improve the analysis of the relationship between various negative events and dependency on mobile phone among adolescents. This study distributed questionnaires to students in ordinary higher education institutions in Jiangsu and Zhejiang regions through online questionnaire links from October 3, 2024 to October 8, 2024, and collected altogether 1399 valid questionnaires. The subjects’ ages spanned from 15 to 25 years, and 121 (8.6%) were 15 years old, 147 (10.5%) were aged 16 years, 129 (9.2%) were aged 17 years, 144 (10.3%) were aged 18 years, 145 (10.4%) were aged 19 years, 177 (12.7%) were aged 20 years, 206 (14.7%) were aged 21 years, 205 (14.7%) were aged 22 years, 54 (3.9%) were aged 23 years, 63 (4.5%) were aged 24 years, 8 (0.6%) were aged 25 years. Of all participants, 742 (53%) were girls. All study participants were volunteers.

### 3.2 Measures

This study sets the argument variable X as a negative event. These events came from the Adolescent Life Events Questionnaire and included punishment, loss, interpersonal pressure, learning stress, adaptation, and other events, with a total of 27 items. However, during the data collection and analysis process, the dimension of “other events” was not effectively filled in by the investigators, so it was omitted. The dependent variable, Y, was mobile phone addiction. To comprehensively evaluate the status of mobile phone addiction among adolescents, this study adopted the mobile phone addiction tendency questionnaire for college students, which covers 16 items in four dimensions: withdrawal symptoms, prominent behaviors, social comfort, and mood changes. In addition, this study introduced two mediating variables, M, namely, M1 personality traits and M2 emotional regulation mode. These variables became the chain intermediary bridge between the X and the Y. There were 40 items in the Big Five Personality Questionnaire and 10 in the Emotional Regulation Questionnaire. The details are as follows.

#### 3.2.1 Adolescent life events questionnaire

The questionnaire adopted the updated Adolescent Life Events Scale (21) by Xin et al. Owing to its targeted design, the questionnaire is highly applicable and accurately reflects the psychological response and stress intensity of adolescents during specific life events. The internal structure of the questionnaire was clear and each event category was equipped with clear scoring criteria and evaluation periods that significantly enhanced the accuracy and reliability of the evaluation results. The scale has 27 items, including seven items on punishment, six items on loss, four items on interpersonal pressure, four items on learning stress, four items on adaptation, and one item on other events. The scale asks participants to review the past 12 months and judge whether the events listed in the questionnaire have affected themselves and their families. If an event occurred, the participants were required to evaluate the most appropriate option on a scale of six, based on how much the event affected their personal lives, the scoring system ranges from 1, indicating no occurrence, to 6, representing extremely severe, with intermediate values of 2 for no effect, 3 for mild, 4 for moderate, and 5 for severe. The greater the score, the higher the degree of influence of the negative events. The Cronbach’s α coefficient of the Adolescent Life Events Questionnaire in this article was 0.951.

#### 3.2.2 College students’ mobile phone addiction tendency questionnaire

This questionnaire adopted the College Students’ Mobile Phone Addiction Tendency Scale (36) compiled by Xiong et al. The scale focuses on investigating mobile phone usage patterns and addictive tendencies among college students and has high comprehensiveness and exploration. It covers multiple dimensions, such as mobile phone use frequency, use time, use scenario, psychological dependence degree, impact on study and life, and other details, and more comprehensively reflects college students’ behavioral habits and psychological characteristics in mobile phone use and its impact on life. The College Students’ Mobile Phone Addiction Tendency Questionnaire was rated on a five point scale, the higher the score, the higher the mobile phone addiction tendency. The Cronbach’s α coefficient of mobile phone addiction tendency scale for college students in this article was 0.959.

#### 3.2.3 Big five personality questionnaire

The questionnaire adopted the abbreviated version of the Chinese Big Five Personality Questionnaire compiled by Wang et al. (37). Through the five broad personality dimensions, the diversity and complexity of human personality traits can be described more comprehensively, providing a more scientific, reasonable, specific, and appropriate description of personality and ability self assessment. The questionnaire comprised a total of 40 items, containing eight items on neuroticism, eight items on conscientiousness, eight items on agreeableness, eight items on openness, and eight items on extroversion. Specifically, six grades were adopted, with one being completely inconsistent and six being completely consistent, seven items were reverse scored. A higher score correlates positively with a greater manifestation of personality traits. Neurotic personality was selected for analysis in the article, and the Cronbach’s α coefficient of the neurotic subscale is 0.930.

#### 3.2.4 Emotional regulation questionnaire

The emotional regulation questionnaire adopted the Emotional Regulation Scale (25) developed by Gross et al. in 2003, who believed that emotional regulation is a dynamic process. In terms of content construction, the emotional regulation scale presents the characteristics of concise and efficient items, and the measurement process is simple and standardized, thereby enhancing the effectiveness and quality of data collection. The scale is composed of cognitive reappraisal and expression inhibition. This division is not only in accordance with the internal logic of emotion regulation theory, it also provides researchers with a more detailed analysis perspective. The cognitive reappraisal dimension consists of six items, while the expression inhibition dimension consists of four items. The emotional regulation scale employs a seven-point point system. The greater the score distribution, the more frequently an individual uses emotional regulation strategies in daily life, thus providing valuable quantitative indicators for researchers. In this article, expression inhibition was selected for analysis, and the Cronbach’s α coefficient of expression inhibition subscale was 0.864.

### 3.3 Statistical analysis

SPSS 27.0 was utilized to the statistical process of the research data. AMOS 23.0 was adopted for mediation effect model analysis. Compared to other research methods, AMOS 23.0 excels in testing whether the research data aligns with the established model, exploring the model, and gradually establishing the most suitable model. This aids researchers in discovering potential patterns and trends within the data. Furthermore, when conducting mediation effect tests, AMOS 23.0 clearly shows the relationship between variables, thereby providing substantial support for a deeper understanding and interpretation of the data.

## 4 Results

### 4.1 Descriptive statistical analysis

Detailed statistics for each variable and its corresponding dimensions, including the mean and standard deviation. The mean score of the overall score of the Big Five Personality test was 138.05, and the average score of the five traits from highest to lowest was neuroticism (29.62), conscientiousness (27.59), extroversion (27.38), agreeableness (27.24) and openness (26.23) (See **Table 1**). Of the personality traits tested, neuroticism was the most significant. Openness was the least common, meaning that the participants generally displayed traits such as anxiety, hostility, depression, high self awareness, impulsiveness, and vulnerability, and relatively low creativity, imagination, and risk taking. Highly neurotic individuals are particularly sensitive to emotional responses, dominated by perceptual thinking, and susceptible to emotional fluctuations. They often express themselves through emotional behaviors, forming a closed personality and reducing social interactions. In terms of emotion regulation strategies, the tested adolescents used cognitive reappraisal strategies more (average score= 22.42) and expression inhibition less (average score=17.83). The influence of various negative events on adolescents varies. When punished, the subjects are most affected (average score 25.49), followed by loss (average score 21.84), adjustment problems (average score 18.43), interpersonal pressure (average score 14.86), and learning stress (average score 14.77). The average score for teenagers’ mobile phone addiction was 52.07. From high to low, the dimension scores were withdrawal behavior (average 19.59), prominent behavior (average 12.97), social comfort (average 9.77), and mood change (average 9.75), indicating that withdrawal was the most common characteristic tested for mobile phone addiction. In the context of mobile phone addiction, withdrawal is a feeling of discomfort caused by not using the phone for a period of time. As modern society continues to develop, information dissemination uses various electronic communication devices. As a convenient communication device, mobile phones help form interpersonal information networks, which are crucial to personal development and the development of society. When the “connection” of mobile phones is lost, many people feel uncomfortable and insecure and even experience physical and psychological discomfort.

**Table 1:** Describes the statistical analysis of each variable.

Questionnaire score	Dimension score	M	SD
Big five personality		138.05	16.09
	Neuroticism	29.62	10.57
	Conscientiousness	27.59	3.79
	Agreeableness	27.24	5.82
	Openness	26.23	10.50
	Extroversion	27.38	5.79
Emotion regulation strategies		40.25	5.98
	Cognitive reappraisal	22.42	5.83
	Expression inhibition	17.83	5.68
Negative events		98.82	28.98
	Punished	25.49	9.46
	Loss	21.84	8.13
	Interpersonal pressure	14.86	5.37
	Learning stress	14.77	5.42
	Adjustment problems	18.43	6.78
	Other events	3.43	1.63
Mobile phone addiction		52.07	18.00
	Withdrawal behavior	19.59	6.95
	Prominent behavior	12.97	4.80
	Social comfort	9.77	3.63
	Mood change	9.75	3.66

### 4.2 Correlation analysis between negative events, neuroticism, expression inhibition, and mobile phone addiction

**Table 2** elucidate r values are all greater than 0, and P values are less than 0.01. It shows that: (1) The remarkable positive correlation between negative events and mobile phone addiction, verifying H1. These data strongly suggest that negative events are an important factor that induces teenagers to become addicted to mobile phones. There were significant positive correlations between various negative events and neurotic traits. And there was a remarkable positive correlation between neuroticism and mobile phone addiction, which can preliminarily predict that neuroticism plays an intermediary role between negative events and mobile phone addiction, that is to say, negative events may raise the risk of mobile phone addiction by enhancing individuals’ neuroticism. (2) A remarkable positive correlation which is between various negative events and expression inhibition. Five categories and expression inhibition, although they had a relatively low correlation coefficient, they reached a statistically significant level. Expression inhibition and mobile phone addiction have a remarkable positive correlation. Thus, it can be predicted that expression inhibition mediates the relationship between negative events and mobile phone addiction. When adolescents face negative events, they may be more inclined to adopt expressive inhibition strategies to deal with emotions, which may further exacerbate their mobile phone addiction.

**Table 2:** Correlation between negative events, neuroticism, expression inhibition, and mobile phone addiction.

	1	2	3	4	5	6	7	8
1. Neuroticism	1.00							
2. Expression inhibition	0.13**	1.00						
3. Punished	0.53**	0.12**	1.00					
4. Loss	0.53**	0.09**	0.73**	1.00				
5. Interpersonal pressure	0.44**	0.09**	0.61**	0.61**	1.00			
6. Learning stress	0.45**	0.12**	0.61**	0.65**	0.56**	1.00		
7. Adjustment problems	0.49**	0.13**	0.63**	0.65**	0.58**	0.58**	1.00	
8. Mobile phone addiction	0.38**	0.08**	0.38**	0.39**	0.33**	0.36**	0.38**	1.00

**p < 0.01.

### 4.3 Analysis of multiple mediation models

To explore the mediating mechanisms of neurotic personality and expression inhibition on negative events and mobile phone addiction, article adopted the AMOS 23.0 software to establish a structural equation mode for a comprehensive analysis, effectively verifying the diverse mediating influences of neurotic personality and expression inhibition. The results were listed in **Tables 3**. According to **Table 3**, negative events have a significant and positive predictive effect on mobile phone addiction (β=0.43), the hazard of mobile phone addiction increases significantly when individual experiences more negative events. At the same time, negative events can significantly positively predict neuroticism (β=0.55), suggesting that negative events have a potentially profound influence on the shaping of individual personality traits, especially neuroticism. **Table 3** also reveals a positive and significant relationship between negative events and expression inhibition (β=0.32). After experiencing negative events, individuals may be more inclined to adopt expression inhibition to deal with emotions, and this emotional regulation mode may further affect the psychological and behavioral states of people. Besides, neuroticism was found to positively and significantly predict mobile phone addiction (β= 0.15) and expression inhibition (β=0.19). Neuroticism was directly associated with mobile phone addiction, it was also indirectly affected by influencing individual emotional regulation (such as expression inhibition). At the same time, expression inhibition was also shown to be a positive predictor of mobile phone addiction (β=0.10), which further emphasizes the importance of emotional regulation in the formation of addictive behaviors. Based on the above analysis, we preliminarily verify hypotheses H2, H3, and H4.

**Table 3:** Regression analysis of variable relationships.

Regression equation		Overall fit index			Significance of regression coefficient	
Result variable	Predictor	R	R2	F	ß	t
Mobile phone addiction	Negative events	0.43	0.18	308.52***	0.43	17.56***
Neuroticism	Negative events	0.55	0.3	606.38***	0.55	24.62***
Expression inhibition	Negative events	0.45	0.21	180.80***	0.32	11.35***
	Neuroticism				0.19	6.49***
Mobile phone addiction	Negative events	0.46	0.21	123.50***	0.30	9.98***
	Neuroticism				0.15	5.35***
	Expression inhibition				0.10	3.76***

***p < 0.001.

**Figure 2:**
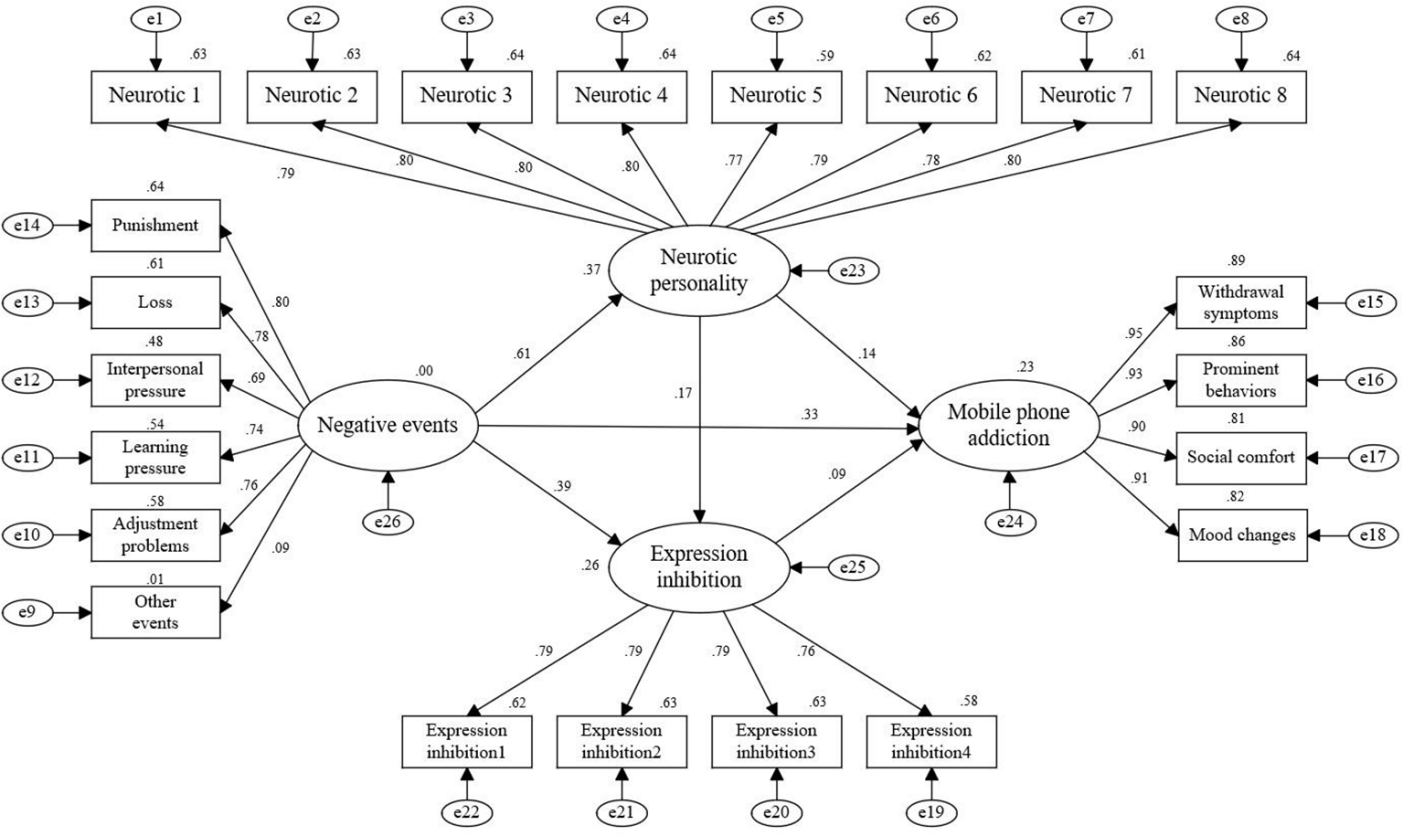
Intermediate effect model of negative events on mobile phone addiction.

SPSS was used to organize the questionnaire survey data, and the six dimensions of negative events were divided into six groups, the four dimensions of mobile phone addiction into four groups, the eight items of neuroticism into eight groups, and the four items of expression inhibition into four groups. Each latent variable was composed of the corresponding observational indicators. AMOS was used to construct a mediating effect model to clearly demonstrate the relationship between each latent variable, as shown in **Figure 2**. Indirect effect values were obtained as shown in **Table 4**. Each fitting index of the structural equation model is as follows: CMIN/DF=1.3, RMSEA=0.01, GFI=0.98, AGF-I=0.98, NFI=0.99, IFI=1.00, TLI=1.00, CFI=1.00, see **Table 5**. Generally, the CMIN/DF value approaching 1 indicates a better fit, a value of less than 5 is acceptable and indicates a good fit of the model. The RMSEA index is less than 0.08, this suggests that the model exhibited a strong fit. The closer the GFI, NFI, IFI, TLI, and CFI indicators are to 1, the better the fit. If these indicators are greater than 0.90, the data support the construct hypothesis, and the structural model has a good fit (38). All fitting indexes of the model meet the statistical requirements, and the fit is good. Path 1: negative events→neuroticism→mobile phone addiction, named M1. Path 2: negative events→expression inhibition→mobile phone addiction, named M2. Path 3: Negative events→neuroticism→expression inhibition→mobile phone addiction, named M3. According to the output results of the structural equation model, the effects of these three pathways are remarkable, with effect values of 0.09, 0.04 and 0.01 respectively. The structural equation model diagram shows that neurotic personality and expression inhibition have a chain intermediary effect between negative events and mobile phone addiction, which means that negative events first affect the neurotic personality traits of individuals, and then eventually affect the behavior of mobile phone addiction through the emotional regulation strategy of expression inhibition. Among them, path 1 has the greatest influence, that is, neuroticism has the most obvious mediating effect on negative life events and mobile phone addiction.

**Table 4:** Mediation effect values of the three pathways.

Parameter	Estimate	Lower	Upper	P
M1 (Neurotic mediation)	0.09	0.04	0.13	Less than 0.01
M2 (Expression inhibition mediation)	0.04	0.01	0.06	
M3 (Chain mediation)	0.01	0.00	0.02	

**Table 5:** Fitting index table of the model.

Index	CMIN/DF	RMSEA	GFI	NFI	IFI	TLI	CFI
Better fit	1-3	≤0.05	≥0.90	≥0.90	≥0.90	≥0.90	≥0.90
Acceptable	3-5	≤0.08	≥0.80	≥0.80	≥0.80	≥0.80	≥0.80
Model	1.3	0.01	0.98	0.99	1.00	1.00	1.00

Based on ideal model fitting, the bootstrap method was employed to establish a 95% confidence interval for assessing and validating the efficacy of the mediation effect. If the interval did not contain 0, it will be deemed significant, otherwise, it was not significant (**Table 6**). Among the total indirect effects produced by neuroticism and expression inhibition, the confidence interval of bootstrap 95% [0.08,0.18] does not include value of zero, indicating that, in addition to the direct influence of negative events on mobile phone addiction, neuroticism and expression inhibition were also significant between negative events and mobile phone addiction. This finding highlights the importance of neuroticism and expression inhibition in linking negative events to phone addiction. Further analysis shows that this intermediary effect includes three indirect effects. In indirect effect 1, negative events→ neuroticis→mobile phone addiction, with effect value of 0.09 and confidence interval of [0.04,0.13]. The indirect effect of this path is significant, indicating a mediating effect of neuroticism reaches a significant level, which means that negative events may increase the risk of mobile phone addiction by increasing the neuroticism of individuals. In indirect effect 2, negative events→expression inhibition→mobile phone addiction, with effect value 0.04 and confidence interval [0.01,0.06]. The indirect effect of this path is significant, indicating that the mediating effect of expression inhibition reaches a significant level and that negative events may prompt individuals to adopt the emotional regulation mode of expression inhibition, thereby increasing the risk of mobile phone addiction. In indirect effect 3, negative events→neuroticism→expression inhibition→mobile phone addiction, with an effect value of 0.01 and a confidence interval of [0,0.02]. The indirect effect of this pathway indicates that neuroticism and expression inhibition have a significant mediating effect on the chain between negative events and mobile phone addiction, which means that negative events may increase an individual’s neuroticism. Individuals are then encouraged to adopt emotional regulation methods of expression inhibition, which ultimately increases the risk of mobile phone addiction. The above conclusions verify hypotheses H2, H3, and H4.

**Table 6:** Analysis of mediating effects.

Effect	Effect size	Boot standard error	Boot 95% CI lower limit	95% CI upper limit
Total indirect effect (Sum of three indirect effects)	0.13	0.02	0.08	0.18
Indirect effects 1 (Neurotic mediation)	0.09	0.02	0.04	0.13
Indirect effects 2 (Expression inhibition mediation)	0.04	0.01	0.01	0.06
Indirect effects 3 (Chain mediation)	0.01	0	0	0.02

## 5 Discussion

Mobile phones have emerged as a crucial gateway point for Internet connections because of features such as functional diversity, real time interaction, and easy operation. This change has promoted the rise of short online video platforms with timeliness and sharing, which greatly meet the learning needs, expression wishes, and social expectations of students, and are favored by teenagers (39). The findings from this research strongly validate Hypothesis 1, indicating a notably positive association between negative events and mobile phone addiction, supporting Hypothesis 2 and elucidating the intermediary function of neurotic personality in the connection between negative events and mobile phone addiction. Hypothesis 3 is also confirmed, revealing that expression inhibition is a mediating mechanism through which negative events significantly affect the occurrence of mobile phone addiction behaviors. Hypothesis 4 is also valid, indicating that negative events, to a certain extent, promote the formation of a neurotic personality, which affects the choice of emotional expression, and ultimately contributes to the onset of mobile phone addiction. Researches provide a crucial basis for understanding the multifaceted pathways involved in mobile phone addiction.

### 5.1 Negative events have a significant positive correlation with mobile phone addiction

In line with previous research, negative events have a remarkable and positive predictive effect on mobile phone addiction (40), at the same time, mobile phone addiction has also been observed to significantly affect the incidence of negative events (16). This study further analyzed and discovered the impact of negative events on mobile phone addiction. When individuals are punished, their understanding of social support is weakened (41). According to dependency theory, when individuals perceive that their mobile phones are a means to obtain social support missing in reality, their attachment mechanism will be activated (42). The social interaction, entertainment, and information acquisition functions of mobile phone play an important role in helping individuals expand or rebuild social networks and obtain online social support, and these functions effectively fill the gap in real needs. However, if an individual becomes overly dependent on this process, it may gradually lead to the formation of addictive behavior problems (8). Cognitive behavioral models explain that social isolation/loneliness is the proximal cause of mobile phone addiction, while psychological factors (e.g depression, social anxiety) are distal factors that indirectly promote mobile phone addiction through the proximal factors (43, 44). In real life, adolescents suffer accidents of loss type, such as the departure of relatives and friends or financial losses, which can cause individuals to have a strong sense of social loneliness (45). Moreover, there are complex mechanisms of action between loneliness, mobile phone addiction, and mental health problems, such as depression (8, 44). Intense sense of loneliness, as a psychological experience, tends to exacerbate individuals’ tendencies towards depression and other psychological problems (46). During this process, individuals tend to use mobile phone networks to avoid reality. Regarding the adaptation problem, situational characteristics theory points out that if an individual cannot quickly adapt to environmental changes, it will lead to negative events such as interpersonal conflict, emotional distress, or learning disabilities (47). The individual’s self defense mechanism will be activated in this situation, and mobile phone use may be their immediate coping strategy to mitigate the impact of negative events (8). The stress interaction theory posits that stress arises from the interaction between individuals and their environment, with the individual’s cognitive appraisal of environmental stressors being the decisive factor. When environmental stress exceeds an individual’s coping ability, stress then occurs (19). Nowadays, students’ knowledge acquisition channels and learning atmospheres have broadened and been optimized, respectively, but have also created corresponding pressure (48). Excessive pressure aggravates college students’ tendency to transformed into addicted to mobile phones because negative stressful events encourage college students to relieve their stress in various ways, such as escaping from reality or seeking social contact and pleasurable experiences. Internet, accessed through mobile phones, has the characteristics of anonymity, disinhibition, and excitability, and the phone becomes an effective tool for students to achieve their stress relief goals (14). The negative events mentioned in this article strengthen adolescents’ motivation to use the mobile phones to varying degrees, and the stronger an individual’s motivation to use the mobile phones, the more serious the problem of out of control mobile phone use (49).

### 5.2 Personality traits (neuroticism) play a partial mediating role between negative events and mobile phone addiction

Neurotic personality partly mediates the relationship between negative events and mobile phone addiction. Individuals who experience negative events are prone to forming a neurotic personality, which leads to mobile phone addiction. Regarding the first pathway of mediating processes, it is concluded that the occurrence of negative events was positively correlated with neurotic personality. As a social stressor, if negative events are not effectively adjusted or adapted by individuals, they will lead to negative emotional experiences. Individuals with neurotic traits have a higher sensitivity to stressors, and after experiencing negative events, they report more interpersonal stress factors to themselves or others, showing a higher degree of negative emotional activation and more painful experiences (50), and even develop personality disorders (51).

Regarding the second pathway of mediating processes, this study showed a positive correlation between neurotic personality and addictive mobile phone behavior. The large number of functional and recreational applications allow mobile phones to be used as an alternative to satisfy personal value needs, attracting highly neurotic individuals to vent emotions, release pressure, and obtain comfort in virtual networks (52, 53). In addition, the biopsychological model of addictive behavior suggests that the susceptibility and maintenance of addictive behavior is a complex causal network composed of social, psychological, and physiological factors (54). Individuals with neurotic personalities who experience negative events often use mobile phone networks to avoid real life problems. This can lead to more risk of mental and physical health problems and problematic behaviors (55).

### 5.3 Emotional regulation mode (expression inhibition) plays a partial mediating role between negative events and mobile phone addiction

The findings of the research suggest that negative occurrence further forecast the inclination towards mobile phones dependency through expression inhibition. Adolescents who have experienced negative events tend to have lower self evaluation and a higher accumulation of negative emotions. This negative self evaluation activates the memory of specific failure events, prompting individuals to frequently revisit these unpleasant experiences (56). In addition, coping with negative events is closely related to the choice of emotion regulation strategies (28), and emotional regulation is a key factor in the event coping stage (57). Although expression inhibition can effectively control the external behavior of emotions, it cannot effectively reduce the frequency of emotional experience (25) and could result in the accumulation of negative emotions over a long period of time (58). Expression inhibition is positively correlated with negative mental health, indicating that it may enhance negative emotions (31). Cultural differences have a profound effect on individuals’ emotional regulation. For example, Western culture tends to encourage emotional expression, whereas Eastern culture, dominated by a social value orientation, advocates for introverted and implicit emotional expressions to maintain harmony in interpersonal relations. In the context of collectivist cultures, emotional exposure may be regarded as a threat to group homogeneity (59).

However, this study found expression inhibition positively correlated significantly with mobile phone addiction, which is consistent with the conclusions of some previous studies (18, 30). However, other scholars have indicated that expression inhibition and mobile phone addiction show negative correlation (60). This may be related to the different interpretations of expression inhibition strategies. In the context of collectivist cultures, some scholars believe that expression inhibition is an appropriate way to maintain interpersonal harmony, which may minimize the issue about mobile phone addiction caused by disharmonious interpersonal relationships (60). However, this article regards expression inhibition as a negative cultural trait that may affect personal physical and mental health, that is, inhibition of expression can not only fail to reduce negative emotional experiences, but may also aggravate the accumulation of negative emotions, resulting in individuals frequently using mobile phones to cope with bad emotions and gradually forming habitual dependence on mobile phones (61). The social compensation hypothesis proposes that a negative living environment prompts individuals to seek online compensation for their lack of offline comfort (20). This compensation effect can be divided into constructive and pathological compensation. The former helps individuals recover normal development, whereas the latter hinders individual growth (62). For individuals who encounter negative events in the real world and choose to express inhibition strategies, frequent Internet access becomes a means of pathological compensation, allowing them to experience “compensatory success” in the virtual world.

### 5.4 Personality traits (neuroticism) and emotional regulation patterns (expression inhibition) play a chain mediating role between negative events and mobile phone addiction

The chain mediation effect aims to reveal the complex mechanism of action between variables, which is characterized by the way that multiple mediating variables are arranged in an intermediary chain in a sequential manner and that independent variables can indirectly affect dependent variables through the intermediary chain (63). Specifically, the chain mediation effect covers multiple mediating links acting simultaneously, as well as ordered mediating processes (64). The results showed that neurotic personality and expression inhibition play multiple mediating roles in the influence of negative events on mobile phone addiction. After encountering negative events, individuals who possess a neurotic personality tend to have a higher probability of adopting negative coping strategies, which improve their negative emotional experience, reduce their satisfaction with real life, drive them to flee to the virtual world and lead to mobile phone addiction behavior. Therefore, neuroticism and expression inhibition may jointly promote the occurrence of mobile phone addiction synergistically. Individuals with high neuroticism have poor emotional stability and need to put more effort into regulating their negative emotions. However, owing to a negative processing bias, they often fail to regulate and resort to maladaptive coping strategies (65). Furthermore, neuroticism can be used to predict the frequency with which individuals use expression inhibition strategies (66). However, this is because neurotic personalities often adopt poor expression inhibition strategies to deal with long term accumulated negative emotions in negative ways (67), such as mobile phone dependence (68). When individuals use mobile phones to achieve happiness, the nervous system activates dopamine, triggering a reward mechanism (69). This experience then evolves into a persistent and stable habit, further exacerbating addictive behavior and trapping individuals in a vicious cycle (40).

## 6 Conclusions

Although this study provides a pathway for how negative events directly or indirectly affect mobile phone addiction through neurotic personality traits and expression inhibition strategies, there are some limitations in this study due to the length of the article and to preserve the study’s focus and depth to the fullest extent possible. Initially, the cross sectional design was employed in the article, which helps reveal the correlation between variables, but determining a causal relationship is not possible. Subsequent studies should consider using an experimental design to observe the specific effects of key variables on mobile phone addiction behavior, thereby enhancing the persuasiveness and reliability of the research conclusions. Second, this study focused on high school and college students. Although this choice is representative, the universality of the conclusions must be considered. Follow up studies should expand the sample scope to cover individuals of different ages, such as children, young adults, and older adults, to comprehensively investigating the varying effects of adverse occurrences on mobile phone addiction in people at different life stages, which will further enrich and improve the existing theoretical system. In addition, the current study did not provide a clear answer as to whether there is a two-way interaction between negative events and mobile phone addiction, that is, whether there is mutual causation. To delve deeper into this complex association, follow up studies should use longitudinal designs to follow the same group of participants over time and observe the dynamic changes to provide a more solid evidence base for understanding the underlying mechanisms of this phenomenon. Finally, the sample statistical population data studied in this article has limitations. Only gender and age are used in this article. In future studies, variables can be further increased to enrich the content of the article.

Despite these limitations, the study found that negative events are not only directly related to the increase in mobile phone addiction tendency, but also further promote over dependence on mobile phone use through the neurotic personality of the individual. At the same time, expression inhibition, as a mode of emotional regulation, also holds significant importance in this process, which serves as another mediating variable between neurotic personality and mobile phone addiction, strengthening this chain reaction. In terms of innovation in research, chain mediation not only shows the super imposed effect of neurotic personality and expression inhibition in the influence path but also highlights the dual mediating effect characteristics of both. Compared with the single simple mediation model in traditional studies, this provides a richer and deeper perspective, which increases the validity of theoretical explanations and the rigor of the empirical evidence. The research has certain enlightening significance, and educators should pay timely attention to students’ mental health, especially those who have experienced negative events, and regularly provide psychological education and counseling. For parents, it is important to establish an effective communication mechanism with their children, promptly identify and resolve negative events they encounter, and prevent them from becoming addicted to their phones due to escaping reality.

## Data Availability

The original contributions presented in the study are included in the article/supplementary material. Further inquiries can be directed to the corresponding author.
